# Diagnostic implication of a circulating serum-based three-microRNA signature in hepatocellular carcinoma

**DOI:** 10.3389/fgene.2022.929787

**Published:** 2022-11-15

**Authors:** Tahira Yousuf, Sadaf Bashir Dar, Sadaf Ali Bangri, Naseer A. Choh, Zubaida Rasool, Altaf Shah, Rafiq Ahmed Rather, Bilal Rah, Gh Rasool Bhat, Shazia Ali, Dil Afroze

**Affiliations:** ^1^ Advance Centre for Human Genetics, Sher-I-Kashmir Institute of Medical Sciences (SKIMS), Srinagar, Jammu and Kashmir, India; ^2^ Department of Immunology and Molecular Medicine, SKIMS, Srinagar, Jammu and Kashmir, India; ^3^ Department of Surgical Gastroenterology, SKIMS, Srinagar, Jammu and Kashmir, India; ^4^ Department of Radio-Diagnosis, SKIMS, Srinagar, Jammu and Kashmir, India; ^5^ Department of Pathology, SKIMS, Srinagar, Jammu and Kashmir, India; ^6^ Department of Gastroenterology, SKIMS, Srinagar, Jammu and Kashmir, India

**Keywords:** circulating miRNA, biomarker, diagnosis, therapeutics, hepatocellular carcinoma

## Abstract

Owing to the diagnostic dilemma, the prognosis of hepatocellular carcinoma (HCC) remains impoverished, contributing to the globally high mortality rate. Currently, HCC diagnosis depends on the combination of imaging modalities and the measurement of serum alpha-fetoprotein (AFP) levels. Nevertheless, these conventional modalities exhibit poor performance in detecting HCC at early stages. Thus, there is a pressing need to identify novel circulating biomarkers to promote diagnostic accuracy and surveillance. Circulating miRNAs are emerging as promising diagnostic tools in screening various cancers, including HCC. However, because of heterogenous and, at times, contradictory reports, the universality of miRNAs in clinical settings remains elusive. Consequently, we proposed to explore the diagnostic potential of ten miRNAs selected on a candidate-based approach in HCC diagnosis. The expression of ten candidate miRNAs (Let-7a, miR-15a, miR-26a, miR-124, miR-126, miR-155, miR-219, miR-221, miR-222, and miR-340) was investigated in serum and tissue of 66 subjects, including 33 HCC patients and 33 healthy controls (HC), by rt-PCR. Receiver operating characteristic curve (ROC) analysis was used to determine the diagnostic accuracy of the prospective serum miRNA panel. To anticipate the potential biological roles of a three-miRNA signature, the target genes were evaluated using the Kyoto Encyclopedia of Genes and Genomes (KEGG) signaling pathway. The serum and tissue expression of miRNAs (Let-7a, miR-26a, miR-124, miR-155, miR-221, miR-222, and miR-340) were differentially expressed in HCC patients (*p* < 0.05). The ROC analysis revealed promising diagnostic performance of Let-7a (AUC = 0.801), miR-221 (AUC = 0.786), and miR-2 (AUC = 0.758) in discriminating HCC from HC. Furthermore, in a logistic regression equation, we identified a three-miRNA panel (Let-7a, miR-221, and miR-222; AUC = 0.932) with improved diagnostic efficiency in differentiating HCC from HC. Remarkably, the combination of AFP and a three-miRNA panel offered a higher accuracy of HCC diagnosis (AUC = 0.961) than AFP alone. The functional enrichment analysis demonstrated that target genes may contribute to pathways associated with HCC and cell-cycle regulation, indicating possible crosstalk of miRNAs with HCC development. To conclude, the combined classifier of a three-miRNA panel and AFP could be indispensable circulating biomarkers for HCC diagnosis. Furthermore, targeting predicted genes may provide new therapeutic clues for the treatment of aggressive HCC.

## Introduction

Globally, hepatocellular carcinoma (HCC) is the third primary cause of cancer-allied mortality and accounts for 90% of primary liver cancers (Yang et al., 2019) (Fattovich et al., 2004; Jemal et al., 2011). Often, HCC is diagnosed at an advanced stage, and due to rapid tumor progression, most HCC patients die early. More than 80% of patients with underlying cirrhosis develop HCC, and, amongst these, only 10% are potentially resectable at the time of detection (Yang and Roberts, 2010; Llovet et al., 2021). The remainders are unresectable because of location, size, or severity of underlying liver disease (Zhang et al., 2014a). Consequently, to improve prognosis, an earlier HCC diagnosis is vital (Bargellini et al., 2014). These imaging techniques are routinely combined with the use of serum tumor markers, such as *α*-fetoprotein (AFP), which is the most commonly used biomarker to detect HCC. Although several western countries have discontinued using AFP due to its poor sensitivity and precision, this protein is still widely used in many Asian countries, including India (Patel et al., 2012). Although the precise molecular mechanisms involved in the development and progression of HCC remain elusive, various risk factors for HCC development are well defined. HBV infection and aflatoxin B exposure are the major risk factors for HCC in high-incidence countries such as Asia and Africa, whereas HCV virus infection, alcohol consumption, and metabolic syndromes are crucial risk factors in low-incidence countries such as Europe, North and South America, and the Middle East. In addition, increased body mass index (BMI) and diabetes, which can lead to non-alcoholic steatohepatitis (NASH), are both substantial risk factors for HCC (Noureddin and Rinella, 2015). This is particularly alarming in view of the rising obesity epidemic in children and adults over the last 25 years (Bhasin Lal et al., 2015). Despite this, around 70–80% of HCC cases emerge from a background of liver cirrhosis, with a median period of 10 years to HCC development (Parkin et al., 2005; Patel et al., 2012). Overall, the poor prognosis and high mortality of HCC are attributable to the underlying cirrhosis, adverse early diagnosis, and paucity of effective late-stage therapy choices (Aleman et al., 2013). This establishes an urgent need to find sensitive markers, especially those correlated with early diagnosis and also for monitoring postoperative recurrence in order to stipulate adequate treatment for HCC. MicroRNAs (miRNAs) are a vital component of the short non-coding RNA family that governs endogenous RNA interference in almost 30% of protein-coding genes by pairing with the 3′ untranslated region (UTR) of mRNA targets, resulting in post-transcriptional and/or post-translational repression (Friedman et al., 2009). miRNAs are known to regulate a broad range of key biological functions, including differentiation, development, proliferation, apoptosis, migration, and invasion in normal cells (Giovannetti et al., 2012). Lately, formidable evidence suggests that miRNAs enact as oncogenes or tumor suppressors, thereby facilitating neoplastic transformation in innumerable cancers, including HCC (Kim, 2005; Acunzo et al., 2015). Additionally, studies have shown the presence of a stable cell-free form of miRNAs in body fluids such as plasma/serum. As a consequence of their inherent stability, miRNAs are considered useful diagnostic biomarkers for noninvasive testing of various types of cancers (Vaz et al., 2015). Numerous studies have recently reported the potential clinical utility of circulating miRNAs in the diagnosis and prognosis of HCC (Arrese et al., 2015). As a result, further research on dysregulated miRNA expression could lead to the identification of novel miRNA biomarkers for HCC. Based on a comprehensive literature review, a panel of ten candidate miRNAs (Let-7a, miR-340, miR-221, miR-222, miR-219, miR-155, miR-126, miR-124, miR-26a, and miR-15a) reported in diverse cancers including HCC were selected for further extensive investigation (Ladeiro et al., 2008; Lawrie et al., 2008; Melo and Esteller, 2011; Cheng et al., 2011; Borel et al., 2012; Anwar and Lehmann, 2015; Arrese et al., 2015; Li et al., 2015; Vasuri and Wang, 2015; Zhuang and Meng, 2015; Dong et al., 2016; He et al., 2018; Wu et al., 2018; Chirshev et al., 2019; Ravegnini et al., 2019; Zhou et al., 2019; Zhu et al., 2020; Liu et al., 2021; Zhang et al., 2021). Prominently, tissue specificity is an essential property of specific biomarkers to perceive cancer progression. Thus, we performed validation in HCC tissues compared to adjacent peritumor sections to confirm the liver-originating serum miRNAs in HCC diagnosis.

## Materials and methods

### Patients and specimen collection

Following stringent inclusion and exclusion criteria, a total of 66 subjects (33 HCC patients and 33 healthy controls) were recruited between January 2018 to December 2020 from the department of Surgical Gastroenterology, Medical Gastroenterology and Radio-diagnosis, Sher-I-Kashmir Institute of Medical Sciences (SKIMS). The details of all participants were collected through a comprehensive questionnaire along with signed informed consent in accordance with approved guidelines of the SKIMS institutional ethics committee (IEC/SKIMS Protocol # RP: 96/2018). The HCC cases enlisted in the study were histopathologically confirmed by liver biopsy or by the radiological findings encompassing abdominal ultrasound (USG), computed tomography (CT) abdomen, magnetic resonance imaging (MRI), or by virtue of elevated serum AFP levels (AFP≥200 μg/ml). Healthy volunteers with no known significant health issues were included as a control group in this research. Additionally, information on clinical features such as BCLC & TNM staging was collected from medical records.

3 ml peripheral blood samples were collected by venous puncture in non-EDTA vacutainers and centrifuged at 4500 x rpm for 10 min at room temperature (RT) to separate the serum fraction for miRNA extraction. Without disturbing the pellet, sera were transferred to fresh collection tubes and stored at −80°C for further analysis. The hemolyzed samples were excluded from the study. Fresh frozen HCC and paired peritumor specimens were collected from HCC patients undergoing surgical resection.

### RNA isolation

The isolation of miRNA from tissue and plasma was done using miRNAeasy Mini Kit (# 1038703, QIAGEN, Germany) and miRNAeasy^®^ Serum/Plasma Kit (# 1071073, QIAGEN, Germany), respectively, by following the manufacturer’s protocol. The concentration and purification of extracted miRNA from tissue and serum/plasma were measured by micro-volume UV-Vis spectrophotometer (Thermo Scientific NanoDrop 2000) at 260 nm wavelength using A_260_/A_280_ ratio. cDNA was synthesized using a universal cDNA kit (# QIAGEN, 21816, Germany). The concentration for each RNA template was adjusted to 5 ng/μL by using nuclease-free water.

### Real-time PCR amplification for the expression of miRNA in tissue and plasma

MiRCURY LNA™ SYBR Green PCR kit (#339346, QIAGEN, United States), commercially designed primers, cDNA, and nuclease-free water were used for amplification of the product. The desired master mix was prepared from the abovementioned reagents in a tube, and the obtained mixture was mixed well by vortexing and spun down. The mixture was dispensed in tubes, and cDNA was diluted (1:60) in nuclease-free water and added to these tubes separately. The tubes were placed in a thermocycler (Rotor-Gene Q MDx, QIAGEN, working on Q-Rex software, Germany) by following reaction conditions as 95°C for 2-min, followed by 40 cycles of denaturation at 95°C for 10-min, annealing at 56°C for 1-min, and melting curve at 60–95°C. U6 was used as an internal reference gene. The ΔΔCt model was used for the relative quantification of RT-PCR data of miRNA.

### Target genes for miRNA signature prediction

From three different miRNA target gene prediction websites, namely, miRTarBase ( http://mirtarbase.mbc.nctu.edu.tw/), TargetScan (http://www.targetscan.org), and miRDB (http://www.mirdb.org/), the database for miRNA prediction was downloaded. The Perl language was used to find miRNA signature targeted genes, which are covered by at least 2 databases. The relationship between miRNAs and target genes was plotted using a Venn diagram and map network.

### Functional enrichment analysis of target genes

The enrichment analysis for predicted genes of potential miRNAs was performed by Gene Ontology (GO) annotation and Kyoto Encyclopedia of Genes and Genomes (KEGG) by using the DAVID database (http://david.abcc.ncifcrf.gov/).

### Statistical analysis

A statistically relevant sample size was calculated by open EPI Statistical Software. All the data are presented as the mean ± SD and/or mean ± SE. The statistical analysis, i.e., “*t*-test”, Spearman correlation (r), univariate logistic regression analysis, stepwise logistic regression analysis and receiver operator curve (AUC), and Kruskal–Wallis test were performed using SPSS version 17.0 (IBM, Chicago, IL, United States), and all figures were generated using GraphPad Prism 5.01 (GraphPad Software, La Jolla, CA, United States). *p* < 0.05 were considered statistically significant.

## Results

### Clinico-pathological characteristics of the study population

The age and gender distribution were indistinguishable in both participating study groups, whereas the liver function parameters of serum bilirubin, ALT, AST, ALP, and AFP were significantly deranged among the participants of the HCC group compared to those of the HC group.

Furthermore, HCC patients had a considerable reduction in platelet counts compared to that in HC patients. However, there were no variations in the serum protein and albumin levels and rates of PT and INR concentration across the two groups. According to CT imaging, 20 of the 33 HCC participants in the study had tumors larger than 5 cm (60%), while 13 had tumors smaller than 5 cm (39%). BCLC 0/A, B/C, and D scores were recorded in 8 (24%), 18 (54%), and 2 (6%) patients, respectively, indicating all patients were in the intermediate to late stages of the disease. In terms of TNM staging, four cases were registered in TNM-I, nine cases in TNM-II, eight cases in TNM-III, and 12 cases in TNM-IV. The demographic features of the study groups, as summarized in Table 1, certify that the subjects recruited in the study are properly classified and accurately represent the allocated group.

**TABLE 1:** Clinico-pathological parameters of study subjects: demographics of the two participating groups. Values are expressed as mean ± SD or depicted as the number of cases (%). Data were analyzed using a non-parametric pair *t*-test (GraphPad Prism 5.0). The statistically significant *p*-value was denoted as < 0.05.

Clinical parameters	Healthy controls (*n* = 33)	Hepatocellular carcinoma (*n* = 33)	*p*-value
Age (Years)	51.68 ± 15.09	56.58 ± 12.29	0.19
Gender (M/F)	23/10	22/11	0.26
S. Bil (mg/dl)	0.95 ± 0.68	1.8293939 ± 1.71	0.01
ALT (U/L)	14.96 ± 8.71	76.263636 ± 72.41	0.001
AST (U/L)	12.36 ± 4.77	141.32 ± 141.35	0.001
ALP (U/L)	94.44 ± 33.64	271.84 ± 231.85	0.001
S. Protein (g/dl)	6.81 ± 1.84	7.12 ± 1.90	0.53
S. Albumin (g/dl)	2.71 ± 0.97	3.096 ± 1.02	0.14
PT (sec)	15.89 ± 3.08	16.8 ± 4.06	0.34
INR (Ratio)	1.23 ± 0.25	1.28 ± 0.31	0.55
Platelet count (*10^9^ per litre)	272.49 ± 93.55	130.15 ± 90.47	0.001
AFP (IU/L)	9.67 ± 4.61	562.77 ± 980.17	0.003
HBsAg positive	0	09 (27%)	—
HCV positive	0	08 (24%)	—
Non-B, Non-C	0	16 (48%)	—
BCLC staging (0-D)	0	02 (6%)	—
0	0	06 (18%)	—
A	0	12 (36%)	—
B	0	11 (33%)	—
C	0	02 (6%)	—
D	—	—	—
TNM staging (I–IV)	0	04 (12%)	—
I	0	09 (27%)	—
II	0	08 (24%)	—
III	0	12 (36%)	—
IV	—	—	—
Tumor size	0	20 (60%)	—
> 5	0	13 (39%)	—
< 5	—	—	—

Abbreviations: SD; standard deviation, S.Bil; serum bilirubin, ALT; alanine amino transferase, AST; aspartate transaminase, ALP; alkaline phosphatase, PT; prothrombin time, INR; international normalized ratio, AFP; alpha-fetoprotein, HbsAg; hepatitis-B surface antigen, HCV; hepatitis C virus, BCLC; Barcelona clinic liver cancer, TNM; tumor, node, and metastasis.

### Screening and validation of candidate miRNAs differentially expressed in HCC

**FIGURE 1:**
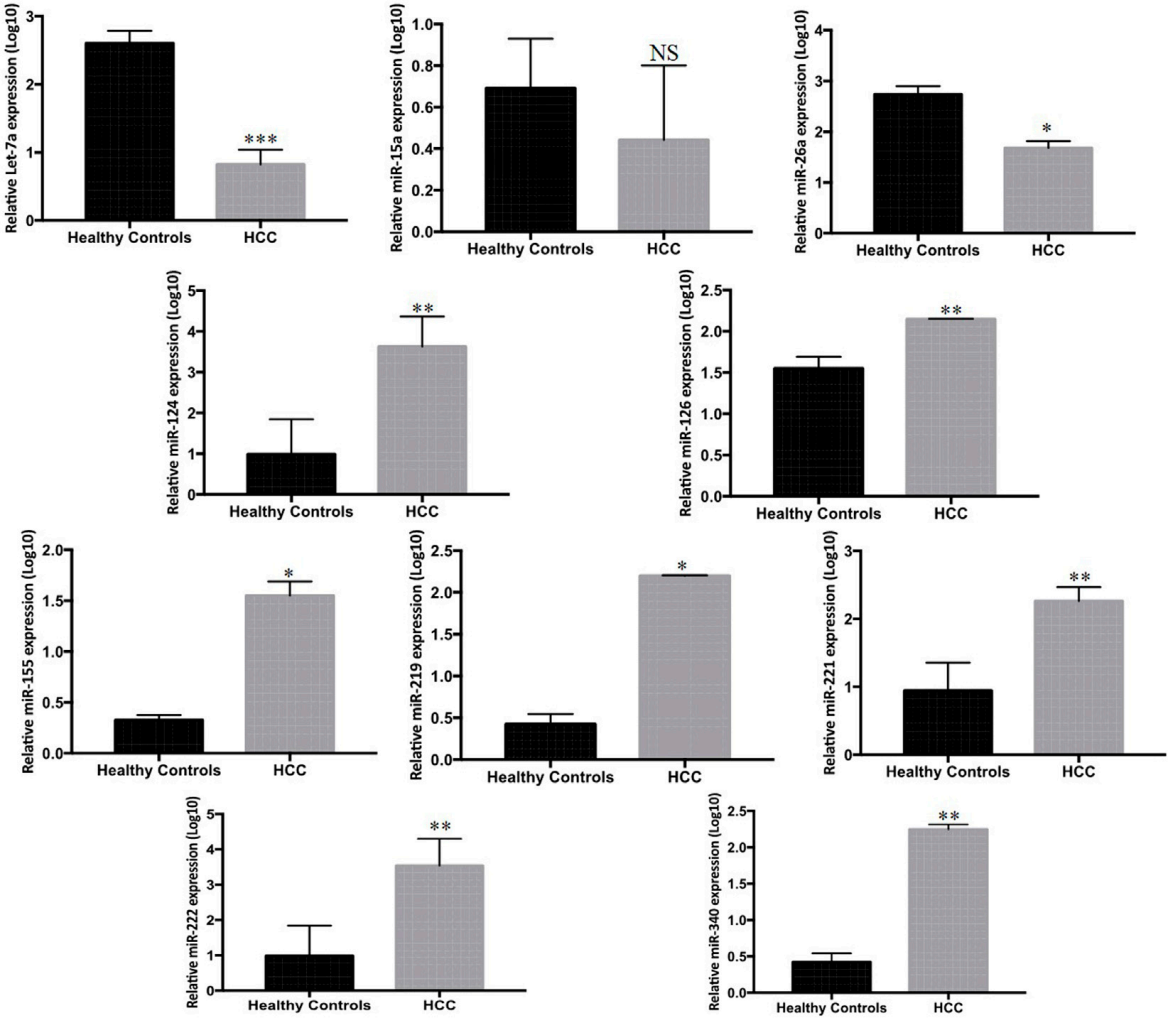
Differential expression of ten candidate miRNAs in sera of HCC patients (*n* = 33) and HC (*n* = 33) measured by Rt-qPCR. The statistical analysis was performed using Mann–Whitney test (GraphPad Prism 5.0). The statistically significant *p*-value was denoted as < 0.05.

Serum expression profiles for the panel of ten miRNAs, entailing (Let-7a, miR-15a, miR-26a, miR-124, miR-126, miR-155, miR-219, miR-221, miR-222, and miR-340) between 33 HCC and 33 HC subjects were observed by real-time qPCR. Our results indicate downregulation of serum miR-221 (*p* < 0.001), miR-340 (*p* < 0.003), miR-126 (*p* < 0.001), miR-219 (*p* < 0.01), and miR-155 (*p* < 0.04) between HCC and control group. Furthermore, Let-7a (*p* < 0.0001), miR-26a (*p* < 0.04), miR-124 (*p* < 0.007), and miR-222 (*p* < 0.005) were downregulated in serum of HCC patients compared to that in HC patients. Furthermore, the expression of miR-15a was non-significant when compared among the groups (Figure 1).

**FIGURE 2:**
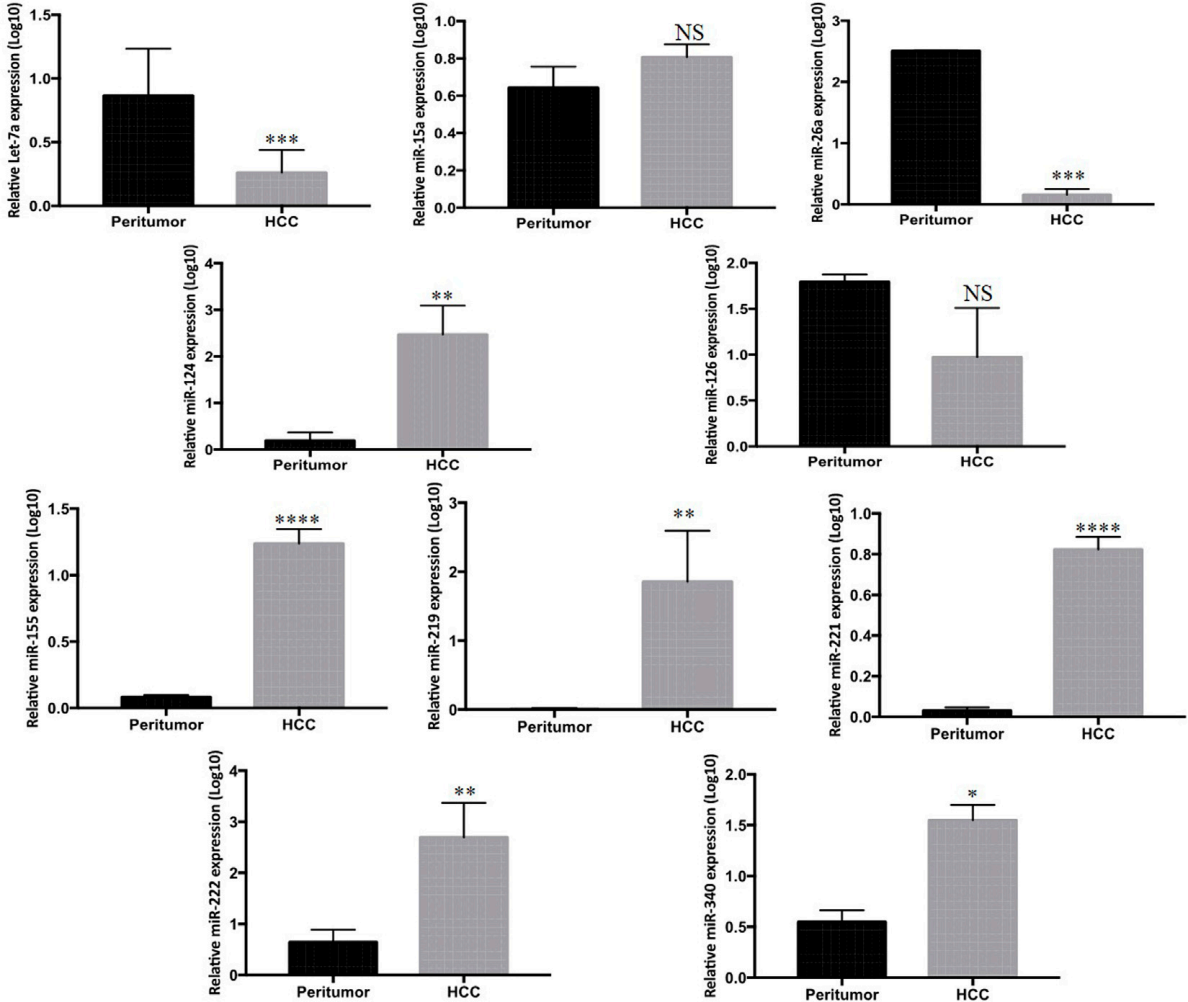
Mean log_10_ expression of ten candidate miRNAs in tumor of HCC patients (*n* = 33) compared to paired peritumor tissue (*n* = 33) determined by Rt-qPCR. The statistical analysis was performed using Mann–Whitney test (GraphPad Prism 5.0). The statistically significant *p*-value was denoted as < 0.05. NS; non-significant.

To further verify the tissue expression profile of the listed miRNAs, the expression levels were measured in 33 tumor and paired peritumor tissues of HCC patients by qPCR. Among the 33 matched samples, miR-221 (*p* < 0.0001), miR-124 (*p* < 0.003), miR-340 (*p* < 0.03), miR-222 (*p* < 0.005), and miR-155 (*p* < 0.0001) exhibited significantly higher expression, while Let-7a (*p* < 0.0007) and miR-26a (*p* < 0.0006) showed marked lower expression in the tumor tissues of HCC patients compared to the peritumor counterparts. Nevertheless, no significant differential expression was found in miR-126, miR-15a, and miR-219 among the groups (Figure 2).

### Clinical significance and diagnostic accuracy of candidate miRNAs in HCC diagnosis

**FIGURE 3:**
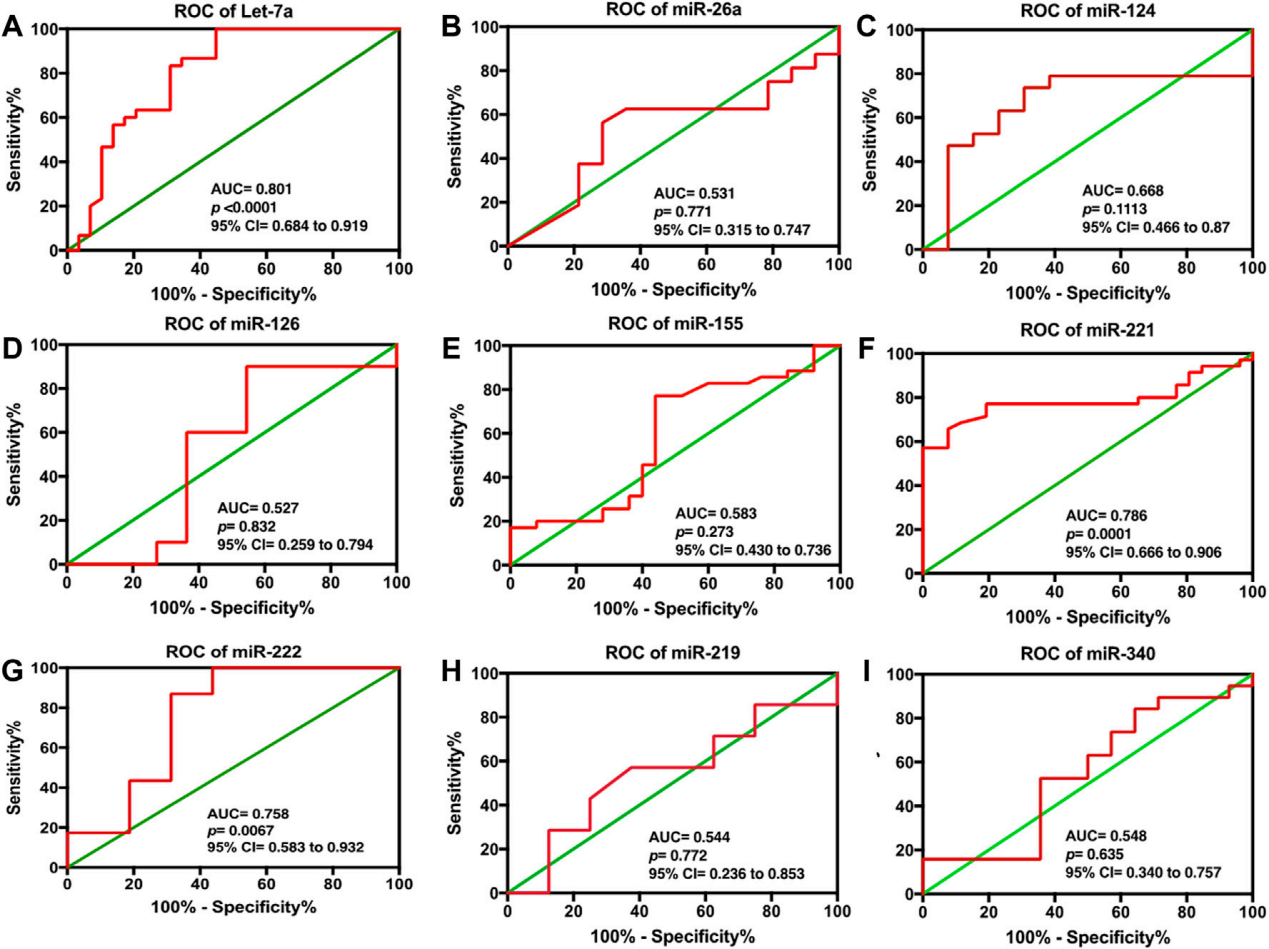
ROC analysis for serum **(A)** Let-7a, **(B)** miR-26a, **(C)** miR-124, **(D)** miR-126, **(E)** miR-155, **(F)** miR-221, **(G)** miR-222, **(H)** miR-219, and **(I)** miR-340 in HCC patients. The statistical analysis was done using GraphPad Prism 5.0.

To validate the discriminatory potential of significantly deranged miRNAs, the area under the curve (AUC) was determined using ROC analysis. Among nine differentially expressed serum miRNAs in HCC patients, Let-7a showed the best diagnostic efficacy with the highest AUC value (AUC = 0.801, 95% CI of 0.684–0.919, and at a cut off <0.9121, sensitivity of 83.33%, and a specificity of 68.97%; *p* < 0.0001), followed by miR-221 (AUC 0.786, 95% CI = 0.666–0.906, cut off <1.626; 228, sensitivity 77.14%, specificity 80.77%, *p* < 0.0001), and miR-222 (AUC 0.758, 95% 229 CI = 0.583–0.932, cut off >0.6096; sensitivity 86.96%, specificity 68.75%, *p* < 230 0.0067). Furthermore, miR-26a (with AUC 0.531), miR-124 (AUC 0.668), miR-340 (AUC 0.548), miR-126 (AUC 0.527), and miR-155 (AUC 0.583) were unable to distinguish HCC patients from HC, as shown in Figure 3.

### Establishment of the predictive three-miRNA panel and to evaluate its efficacy for diagnosis

Based on the univariate logistic regression model, the levels of Let-7a (*p* < 0.001), miR-221 (*p* < 0.0001), and miR-222 (*p* < 0.004) depicted considerable variation among HCC patients and healthy controls and, thus, were accordingly included in the further verification, as shown in Table 2.

**TABLE 2:** Establishment of the predictive three-miRNA panel and to evaluate its efficacy for diagnosis: univariate logistic regression analysis of candidate miRNAs. The statistical analysis was done using GraphPad Prism 5.0.

miRNAs	Estimate	Std. Error	*p-*value
hsa-Let-7a-5p	2.426	0.997	<0.001
hsa-miR-221-3p	-3.175	1.105	<0.0001
hsa-miR-222-5p	5.098	1.968	<0.004

**FIGURE 4:**
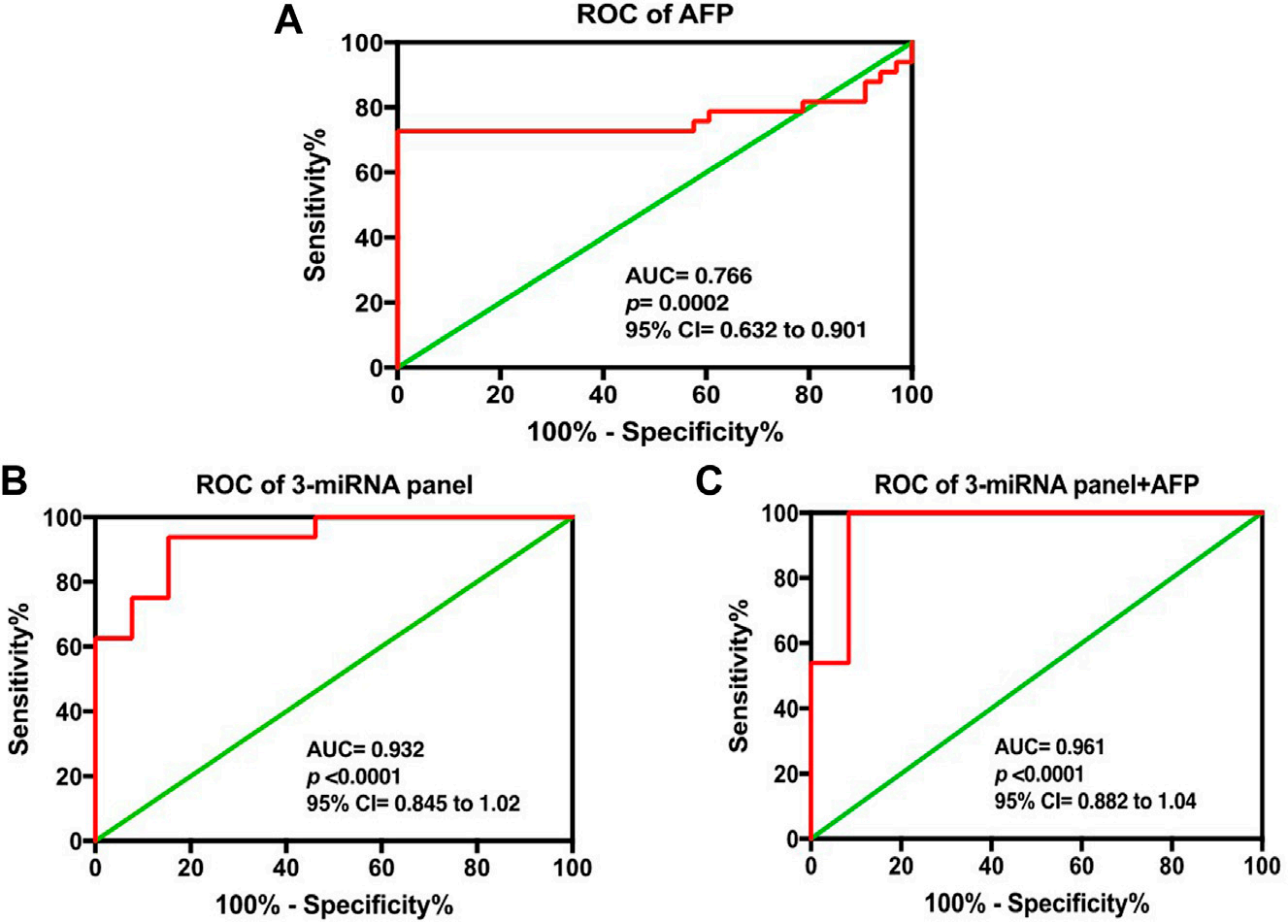
ROC analysis based on logit model for serum **(A)** AFP alone, **(B)** three-miRNA panel alone, and **(C)** combination of three-miRNA with AFP in discriminating HCC from. HC. The statistical analysis was done using GraphPad Prism 5.0.

Altogether, the three chosen miRNAs were found to be significant predictors for HCC, as shown in Table 3. To assess the predicted likelihood of being diagnosed with HCC, the logit model [logit (p) = 0.997 + 3.641 × (Let-7a) -2.985 × (miR-221) + 5.438× (miR-222)] was used to construct the ROC curve. Our analysis affirmed that the AUC of the three-miRNA panel was 0.932 (*p* < 0.0001), with a sensitivity and specificity of 93.75% and 84.62%, respectively, as shown in Figure 4.

**TABLE 3:** Stepwise logistic regression analysis of candidate miRNAs. The statistical analysis was done using GraphPad Prism 5.0.

miRNAs	Estimate	Std. Error	*p*-value	Optimal cut-off	Diagnostic performance
Intercept	0.997	0.245	<0.0001	<7.895	AUC = 0.932 CI = 0.845–1.02 Sensitivity = 93.75% Specificity = 84.62%
Let-7a	3.641	1.420	0.004		
miR-221	−2.985	1.088	0.0005		
miR-222	5.438	2.721	0.0132		

### Compounding accuracy of three-miRNA signature and AFP for the diagnosis of HCC

A step-wise logistic regression model was utilized to analyze the performance of the three-miRNA panel in combination with AFP in discriminating HCC from HC. ROC analysis revealed that the combination of miRNAs (Let-7a, miR-221, and miR-222) had a significant superiority (AUC = 0.961; 95% CI = 0.882), than each miRNA separately; *viz* let-7a (AUC = 0.801, 95% CI = 0.684–0.919), miR-221 (AUC = 0.786, 95% CI = 0.666–0.906), and miR-222 (AUC = 0.758, 95% CI = 0.583–0.932) and AFP alone (AUC = 0.766; 95% CI = 0.632–0.901) in discriminating patients with HCC from those with HC. Additionally, our data suggest that the diagnostic efficacy of Let-7a and miR-221 alone was superior to that of AFP, while miR-222 had a diagnostic efficiency equal to that of AFP, as shown in Table 4.

**TABLE 4:** Stepwise logistic regression analysis of candidate miRNAs in combination with AFP. The statistical analysis was done using GraphPad Prism 5.0.

miRNAs	Estimate	Std error	*p-*value	Optimal cut-off	Diagnostic performance
Intercept	−1.372	2.90	0.0071	—	—
hsa-Let-7a-5p	0.523	0.253	0.0506	—	—
hsa-miR-221-3p	0.890	0.482	0.0700	>6.38	AUC = 0.961
hsa-miR-222-	−0.849	0.320	0.0004	—	CI = 0.882
5p	—	—	—	—	Sensitivity = 100%
AFP	0.107	0.038	0.0301	—	—
				—	Specificity = 91.67%

### Expression of three-miRNA panel signature in viral etiology (HBV/HCV)-related HCC

**FIGURE 5:**
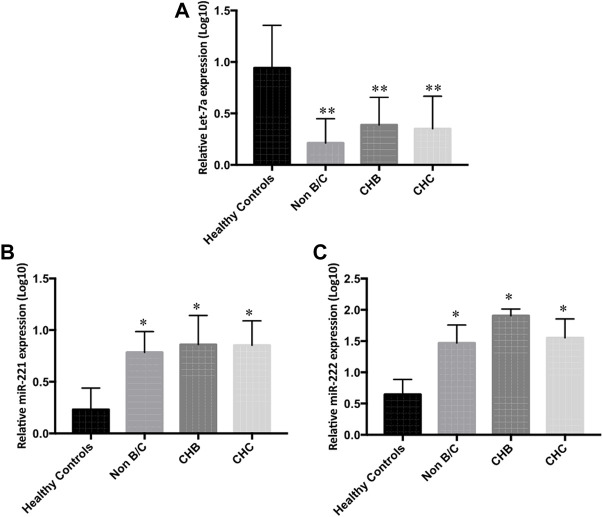
Differential serum expression levels of **(A)** Let-7a, **(B)** miR-221, and **(C)** miR-222 in non-B/C (non-hepatitis B and non-hepatitis C), CHB (chronic hepatitis B), and CHC (chronic hepatitis C) groups of patients compared to peritumor tissue were measured by rt-qPCR. The statistical analysis was done using Mann–Whitney test (GraphPad Prism 5.0). The statistically significant *p*-value was denoted as < 0.05. NS; non-significant.

**FIGURE 6:**
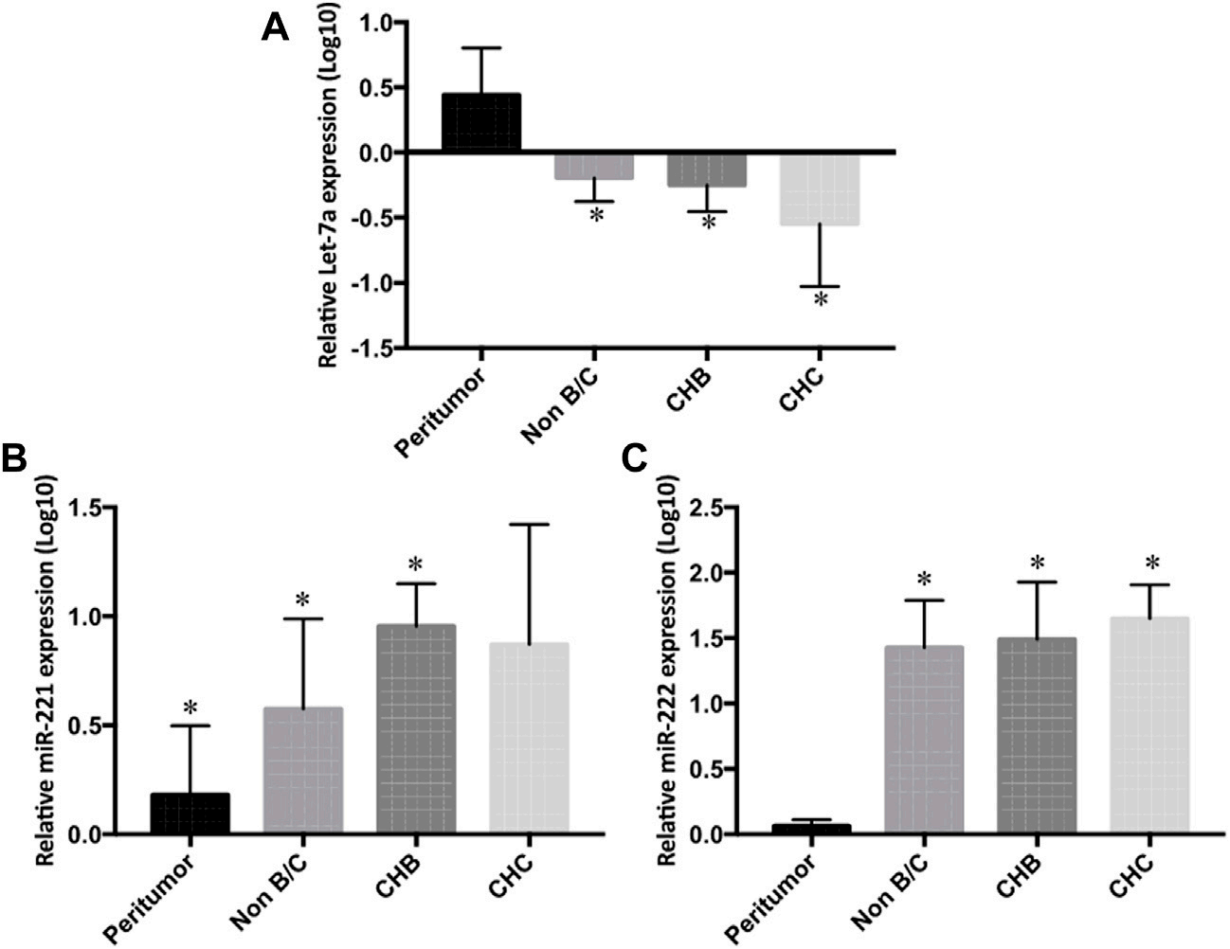
Differential tissue expression of **(A)** Let-7a, **(B)** miR-221, and **(C)** miR-222 in non-B/C (non-hepatitis B and non-hepatitis C), CHB (chronic hepatitis B), and CHC (chronic hepatitis C) groups compared to peritumor tissue were measured by rt-qPCR. The statistical analysis was done using Mann–Whitney test (GraphPad Prism 5.0). The statistically significant *p*-value was denoted as < 0.05. NS; non-significant.

In a further detailed analysis, we investigated serum Let-7a, miR-221, and miR-222 expression profiles in patients with hepatitis B- and hepatitis C-associated HCC. Using the non-parametric Kruskal–Wallis test, we examined that the expressions of three studied miRNAs were significantly different in the sera and tissue of hepatitis B infected, hepatitis C infected, and non-viral groups compared to the healthy controls and peritumor samples (*p* < 0.05), respectively. No significant difference was found in miRNA levels among hepatitis B infected, hepatitis C infected, and non-viral groups (Figures 5 and 6).

### The correlation of serum Let-7a, miR-221, and miR-222 expression and clinical indicators of patients with HCC

Next, to investigate whether an altered expression profile of serum Let-7a, miR-221, and miR-222 is indicative of HCC diagnosis, the clinical correlation was measured. No significant association was found between age, gender, S.Bil, S.ALT, S.AST, S.ALP, S.protein, S.albumin, PT, INR, S.AFP, and BCLC/TNM staging and the expression of Let-7a, miR-221, and miR-222.

#### Prediction of target genes for the three miRNAs

**FIGURE 7:**
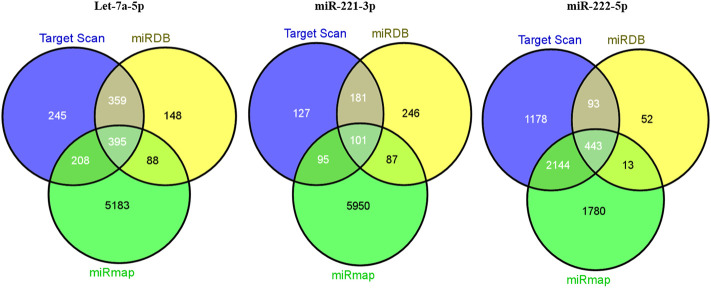
Venn diagram for target genes prediction for miRNA signature.

The target genes regulated by the three miRNAs were predicted by three databases. To further enhance the reliability of the bioinformatic analysis, the overlapping target genes were identified. The results indicated that 395, 101, and 443 overlapping genes were identified for Let-7a, miR-221, and miR-222, respectively, by the three databases mentioned previously, which were shown using a Venn diagram (Figure 7).

**FIGURE 8:**
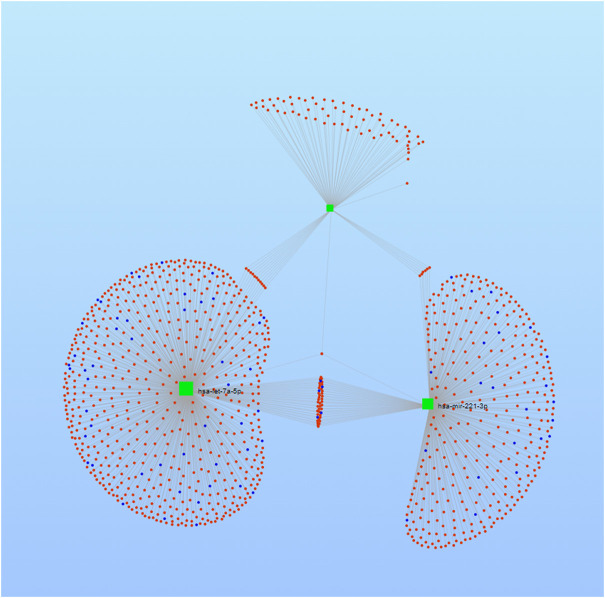
Network map of miRNA target genes.

A total of 1207, 990, and 5874 for hsa-Let-7a-5p, 504, 615, and 6233 for hsa-miR-221-3p, and 3858, 601, and 4380 for hsa-miR-222-5p target genes were predicted using three databases *viz.* TargetScan, miRDB, and mirMap, respectively. To clarify whether the target genes of these miRNAs are likely to participate in the progression of HCC, the intersection of target mRNAs for downregulated miRNAs (hsa-Let-7a-5p) and upregulated mRNAs and target mRNAs for upregulated miRNAs (hsa-miR-221-3p and hsa-miR-222-5p) and downregulated mRNAs were taken. The results were performed on a total of 1,034 genes, including 780 upregulated genes and 254 downregulated genes, respectively. The network between the three miRNAs and their 1034 target genes is shown in Figure 8.

#### Functional enrichment analysis of target genes associated with HCC

**FIGURE 9:**
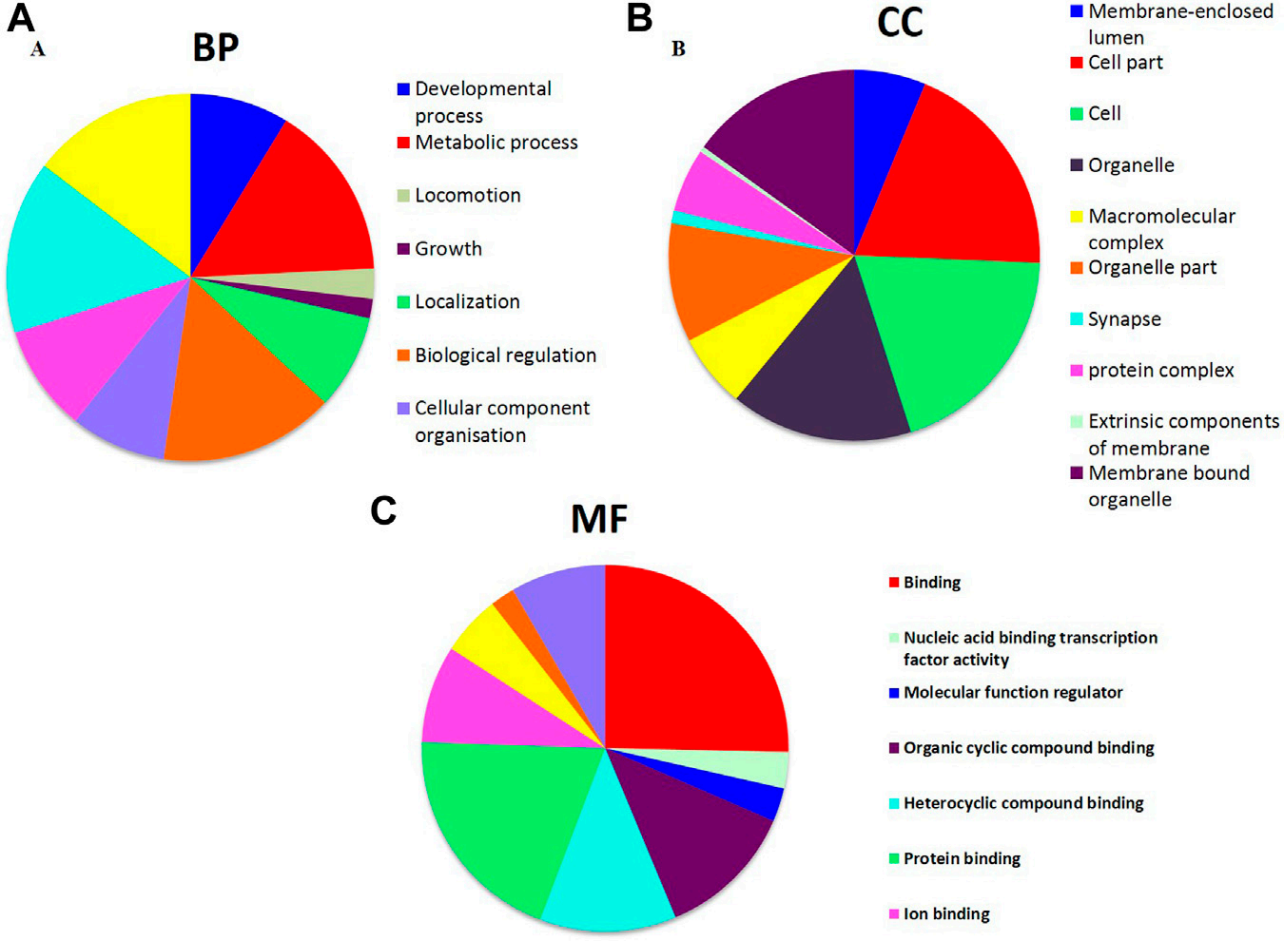
GO annotation for the predicted target genes of potential differentially expressed miRNAs. **(A)** Top ten enriched biological process (BP) for target genes of miRNAs. **(B)** Top ten enriched cellular component (CC) for target genes of miRNAs. **(C)** Top ten enriched molecular function (MF) for target genes of miRNAs.

**FIGURE 10:**
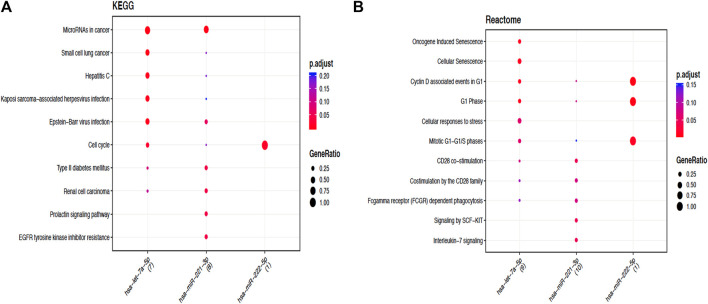
Functional enrichment analysis of target genes associated with HCC. **(A)**. Dotplot of the KEGG signal pathway showing the counts of genes. Functional enrichment analysis of target genes associated with HCC. **(B)** Reactome of the KEGG signal pathway showing the “pathway–gene” network.

In order to perceive the structured features and biological interpretation of the 1034 predicted target genes, the Gene Ontology (GO) annotation was conducted using the AVID database. The results for the three listed categories of GO, comprising cellular component (CC), biological process (BP), and molecular function (MF), were demonstrated in a pie chart. Among the top ten particulars of target genes, BP analysis mostly includes metabolic process, biological regulation, and organic substance metabolic process. Furthermore, CC analysis mainly contained cell, cell part, and organelle, while MF analysis mainly contained binding, protein binding, and organic cyclic compound binding (Figure 9). The results of KEGG pathways enrichment analysis about the target genes associated with HCC were mainly enriched in the microRNAs in cancer, oncogene induction senescence, pancreatic adenocarcinoma pathway, Fas ligand pathway, stress induction of heat shock proteins regulation, and hepatitis C and HCC pathway (Figure 10 and Supplementary Figures S1 and S2).

## Discussion

HCC is the fifth most prevalent malignancy and the second dominant cause of cancer-related mortality worldwide, with a 5-year survival rate of barely 5%, making it the third most lethal malignancy (Huang et al., 2021). The dismal prognosis of HCC is mostly owing to the late diagnosis of this disease. Hence, early detection of HCC can facilitate effective treatment and minimize disease mortality. Currently, serum levels of liver enzymes such as ALT, AST, glutamyl transpeptidase (GGT), and AFP are being utilized as biomarkers to assess liver damage and therapy response (Gowda et al., 2009). Even so, ALT, AST, and alkaline phosphatase levels might also be raised as a result of muscle and bone injury (Giannini et al., 2005). In addition to this, numerous novel serum indicators, such as DCP and AFP-L3, have been found to have higher diagnostic efficacy for HCC, yet these have to be widely verified in routine clinical practice (Zhou et al., 2021). This raises concerns about the specificity of existing diagnostic markers utilized in clinics, necessitating a deeper knowledge of the present liver-specific biomarkers. Furthermore, since HCC is frequently identified at an advanced stage, resulting in severe liver damage and restricted treatment choices, new sensitive biomarkers should be sought to overcome early detection.

Recently, miRNAs have emerged as novel non-invasive biomarkers for the diagnosis and prognosis of various human cancers, including HCC. Importantly, a number of studies have documented the tissue- or organ-specific expression of microRNAs, suggesting the high specificity of microRNAs as biomarkers. Since the first report on the use of microRNA as potential diagnostic biomarkers by Lawrie et al. in 2008 on large B-cell lymphoma, the notion has been pursued in a number of studies involving various other cancer types, including the potential use of circulating miRNAs as biomarkers for HCC (Yang et al., 2019). An extensive and considerable number of studies have reported dysregulation of microRNAs during the initiation and progression of HCC. These studies were largely based on comparisons between malignant, benign, and pre-cancerous lesions compared with a healthy liver or adjacent peritumoral tissues. The approach of direct measurement of tissue microRNAs is challenging as invasive biopsy or surgery is required for collecting tissue samples. Conversely, circulating microRNAs provide an alternative non-invasive approach to be used as tumor biomarkers. Notably, the level of liver microRNAs has been found to correlate with serum microRNA levels in many studies (Shao et al., 2019; Mohamed et al., 2020). One of the major advantages of mature microRNAs as biomarkers is their stability in body fluids such as serum, plasma, urine, saliva, and cerebrospinal fluid over a wide range of temperatures (Ting et al., 2020). Mature microRNAs are complexed with AGO2 or other argonaute proteins, thereby augmenting their stability (Yang et al., 2018). Liver microRNAs may be secreted into the circulation passively through apoptosis and necrosis as ribonucleoprotein complexes or may be transported within viral particles such as HBV surface antigen (HBsAg) or enclosed within exosomes/microvesicles (Shimizu et al., 2010; Yang et al., 2018; Singal et al., 2020). Therefore, the capability to measure microRNA levels secreted in the serum might stipulate a way to estimate microRNA activity in the liver.

In recent years, several miRNAs have been proposed to exhibit high diagnostic accuracy in discriminating patients with HCC from healthy controls. Studies have shown serum miR-143 and miR-215 as promising biomarkers for hepatocellular carcinoma (Zhang et al., 2014b). Xie et al. found that miR-101 could significantly differentiate HBV-HCC from HBV-associated liver cirrhosis (Moeini et al., 2012). Another study reported serum miR-150 as a biomarker for the diagnosis and prognosis of HCC patients (Joyce and Pollard, 2009). Li et al. classified miR-18a as a potential biomarker for screening hepatitis B virus-related HCC (Hatziapostolou et al., 2011). (El-Ahwany et al., 2019) So far, the conclusions on the use of serum miRNAs as non-invasive biomarkers have been contradictory, and efforts to develop a less-invasive approach using serum miRNA combination have met limited success. Thus, in the present study, we hypothesized that inclusion of additional markers, such as miRNAs, might further increase the value of the HCC diagnosis investigations.

Consequently, based on the comprehensive literature review, a panel of ten miRNAs (Let-7a, miR-221, miR-26a, miR-124, miR-222, miR-340, miR-126, miR-15a, miR-219, and miR-155) that were reported in diverse cancers together with HCC was chosen for the study.

In order to examine the regulation of differentially expressed miRNAs in hepatocellular carcinoma, regulation of the panel of ten miRNAs (Let-7a, miR-221, miR-222, miR-26a, miR-124, miR-340, miR-126, miR-15a, miR-219, and miR-155) selected on the basis of literature and *In Silico* analysis was carried out in HCC patients. Our results indicated that miR-221, miR-340, miR-126, miR-219, and miR-155 were upregulated, whereas Let-7a, miR-26a, miR-124, and miR-222 were downregulated in HCC patients compared to HC. However, the expression of miR-15a was non-significant when compared among the groups (Figure 1).

Correspondingly, while investigating the tissue levels, we found the high expression of miR-221, miR-124, miR-340, miR-222, and miR-155 and low expression of Let-7a and miR-26a in the tumor tissue of HCC patients compared to their peritumor counterparts. Nevertheless, the expression of miR-126, miR-15a, and hsa-miR-219 was found to be non-significant (Figure 2).

Furthermore, our data indicated that among the nine differentially expressed miRNAs, Let- 7a showed the best diagnostic efficacy, followed by miR-221 and miR-222, whereas miR-26a, miR-124, miR-340, miR-126, and miR-155 were unable to distinguish HCC patients from HC patients (Figure 3).

Subsequently, a step-wise logistic regression model was utilized to analyze the performance of the three-miRNA panel in combination with AFP in discriminating HCC from HC. We found that the combination of Let-7a, miR-221, and miR-222 had a significant superiority over each miRNA separately and AFP alone in distinguishing HCC patients from HC. Additionally, our data also suggested that the diagnostic efficacy of Let-7a and miR-221 alone were superior to that of AFP, while miR-222 had a diagnostic efficiency equal to that of AFP (Figure 4).

Subsequently, with the aim to understand the interactome of predicted target genes for Let-7a, miR-221, and miR-222, a total of 1,034 intersecting genes predicted by three bioinformatic tools were used to construct the miRNA-mRNA interaction network (Figure 8). Among the top ten particulars of target genes, BP analysis mostly includes metabolic process, biological regulation, and organic substance metabolic process. Furthermore, CC analysis mainly contained cell, cell part, and organelle, while MF analysis mainly contained binding, protein binding, and organic cyclic compound binding (Figure 9). KEGG pathway enrichment analysis revealed that the genes were mainly enriched in the microRNAs in cancer, oncogene induction senescence, pancreatic adenocarcinoma pathway, and hepatitis C and HCC pathway, indicating possible crosstalk of miRNAs in cancer with HCC (Figure 10 and Supplementary Figures S1 and S2).

## Conclusion

In conclusion, we identified three miRNAs; Let-7a, significantly expressed at a lower level, and miR-221 and miR-222, significantly expressed at higher levels, in HCC patients compared to HC volunteers. Based on the ROC analysis, we propose that these three miRNAs can be feasible noninvasive HCC biomarkers. Notably, the panel of the three miRNAs (Let-7a, miR-221, and miR-222) showed the best diagnostic performance than a single miRNA. In a profound analysis, we found that plasma Let-7a, miR-221, and miR-222 expression profiles were common to patients with hepatitis B- and hepatitis C-associated HCC irrespective of the underlying etiology. It is, thus, worthwhile to validate the use of the three-miRNA panel reported in our study for second-line testing in a larger cohort of patients and elucidate the underlying mechanism of the deregulation of circulatory miRNAs. Furthermore, the possible function is inferred by predicting the model for target genes, which improves our understanding of HCC carcinogenesis and progression. To our knowledge, this is the first clinical study that delineated the role of a three-miRNA-panel (comprising Let-7a, miR-221, and miR-222) in the non-invasive diagnosis and plausible therapeutic management of HCC patients. Given the critical role of Let-7a, miR-221, and miR-222 in HCC development, this three-miRNA-panel may improve risk prediction and furnish new insights into the pathological mechanism of HCC development. Together with AFP, this three-miRNA-panel reposes better efficacy in discriminating HCC patients from healthy controls (HCs) than the existing miRNA-based biomarkers. This study may also help in the identification of novel targets for the therapeutic intervention of HCC and may provide a rationale for improving existing cancer therapies, prominently driven by the fact that both hepatitis B virus (HBV) and hepatitis C virus (HCV) infections result in chronic hepatitis paramounting to the HCC development. Furthermore, the expression of miRNAs in response to HBV and HCV infection varies dramatically between the two viruses. Henceforth, the early detection and rapid development of new therapeutic antivirals for HCC necessitate the discovery of novel miRNA biomarkers common to the array of all etiologies.

## Data Availability

The original contributions presented in the study are included in the article/Supplementary Material; further inquiries can be directed to the corresponding author.
