# Supplementary figures and images for: Diagnostic implication of a circulating serum-based three-microRNA signature in hepatocellular carcinoma
Source: Front Genet. 2022 Nov 15;13:929787. doi: 10.3389/fgene.2022.929787 (PMC9705795; doi:10.3389/fgene.2022.929787)

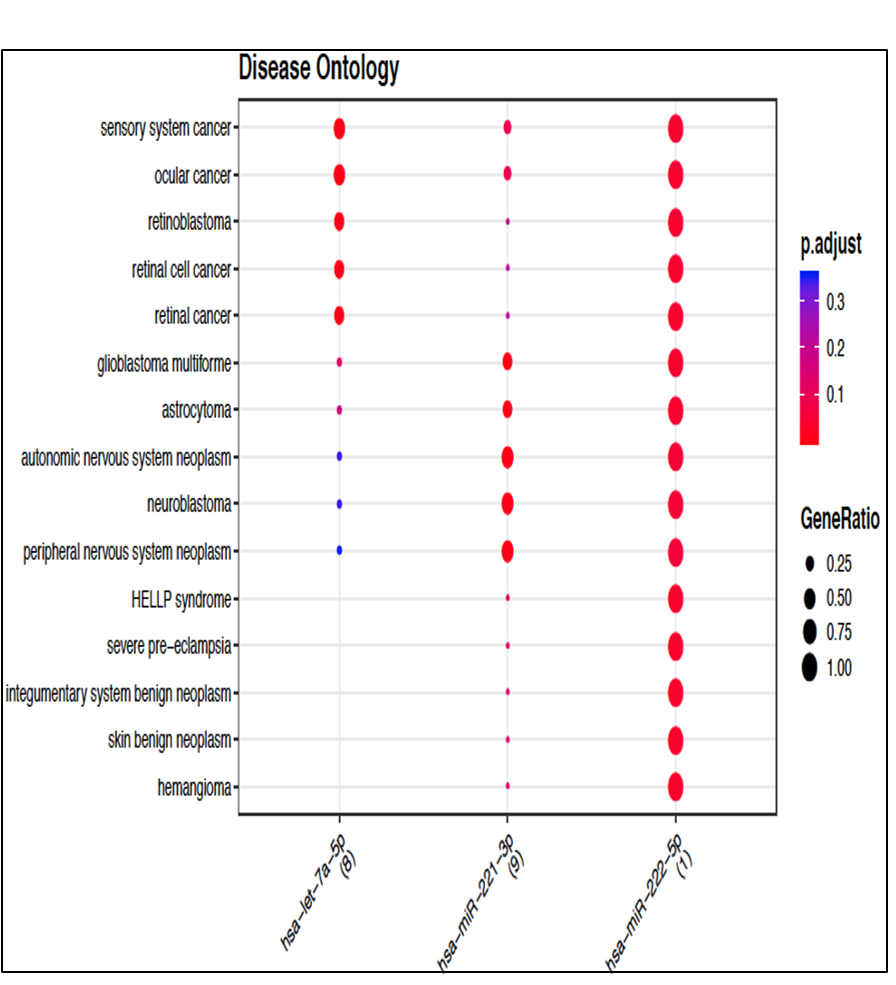

Supplement: Supplementary file 1 [file Image2.tif]

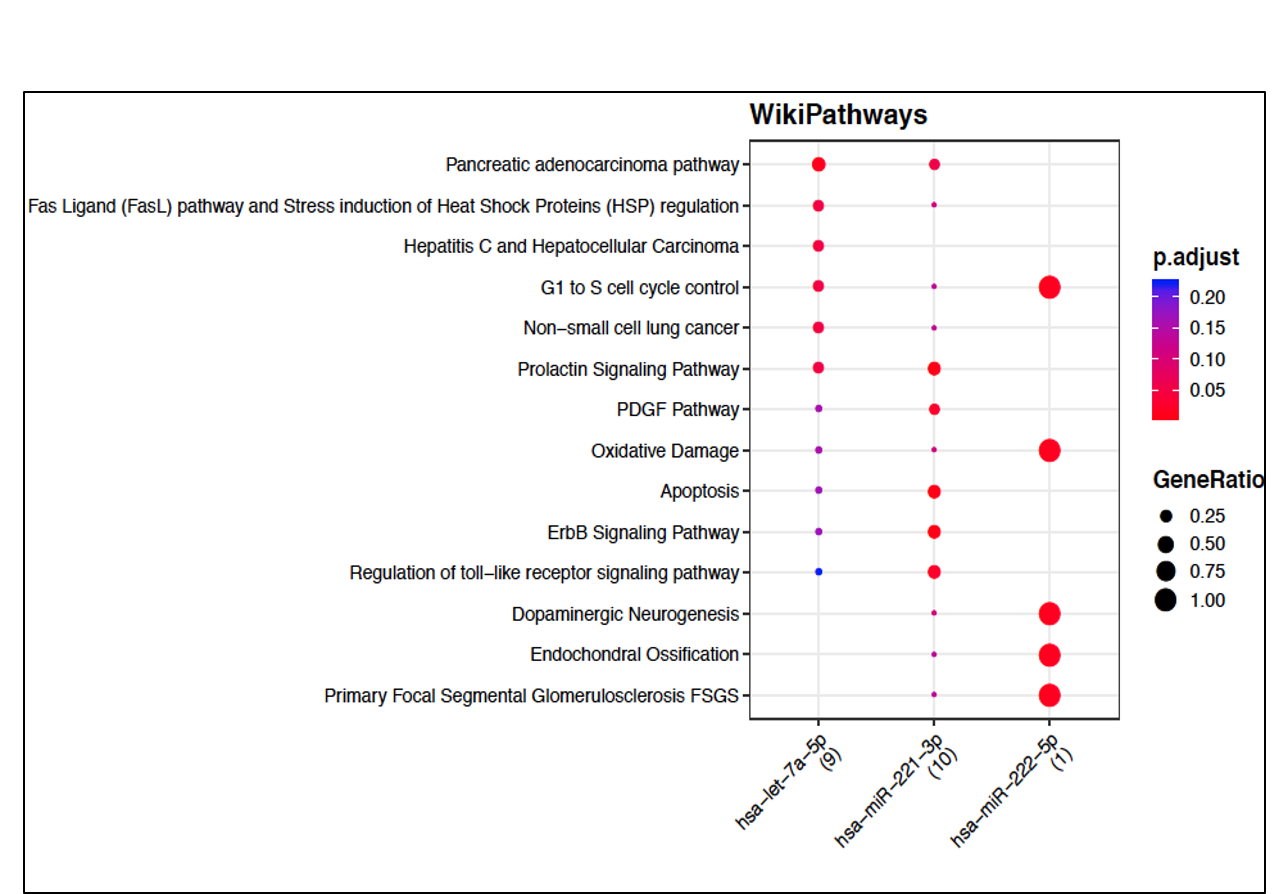

Supplement: Supplementary file 2 [file Image1.tif]
